# Supplementary material for: Are carbon emissions trading and green financial instruments synergistic? -Comprehensive quantitative research based on content analysis
Source: PLoS One. 2024 Mar 7;19(3):e0298601. doi: 10.1371/journal.pone.0298601 (PMC10919601; doi:10.1371/journal.pone.0298601)
Supplement: S1 Data — (ZIP) [file pone.0298601.s001.zip › Data/地级市Z_F工作报告.zip!/╔╜╢1⁄2/╢1⁄2╙¬╩╨/└┤╫╘╣1⁄2╓┌║┼í╛┬φ┐╦╩2╛▌═°í┐_2004.docx]

各位代表：

现在，我代表市人民政府向大会作政府工作报告，请予审议，并请各位政协委员和其他列席人员提出意见。

一、2003年政府工作回顾

2003年是新一届政府工作的开局年，是全面建设小康社会的起步年。一年来，全市人民高举邓小平理论和“三个代表”重要思想伟大旗帜，以党的十六大、十六届三中全会精神为指导，在省委、省政府和市委的正确领导下，同心同德，奋力拼搏，胜利完成了市五届人大一次会议确定的各项任务，实现了开好局、起好步的目标。去年市委工作会议确定了“一个总目标”、争取“两个率先”、实现“三个显著提高”的目标任务和“十突出、十加快”的工作思路，全市上下进一步掀起了解放思想、干事创业、加快发展的热潮，呈现出竞相发展、蒸蒸日上的可喜局面，经济社会发展进入了一个新的阶段。

(一）国民经济持续快速协调健康发展。初步统计，2003年全市国内生产总值完成691亿元，比上年增长16%，其中地方311亿元，增长21.6%。农林牧渔全面丰收，农业结构调整迈出了较大步伐，优势产业发展迅速，农业产业化、标准化水平不断提高，农业增加值增长7.9%。工业发展速度加快，运行质量明显提高。全市规模以上工业增加值增长28.1%，其中地方增长41%；地方规模以上工业实现利税和利润分别增长43.9%和49%，胜利油田经济效益指标创历年最好水平。高新技术产业产值占全市规模以上工业产值的比重提高了2.1个百分点。城乡市场繁荣活跃，全市社会消费品零售总额增长15.7%。固定资产投资增势强劲，全社会完成固定资产投资338.6亿元，增长40.6%，其中地方218.3亿元，增长75.9%。民营经济蓬勃发展，个体工商户达到4.4万户，民营企业发展到7406家，上缴税金增长40.1%。县域经济发展活力增强，广饶县进入了全省30强。财政收入大幅度增长，境内财政总收入达143.4亿元，其中全市地方财政收入23亿元，增长26.7%。金融运行良好，各项存款余额4958亿元、贷款余额296.7亿元，分别比年初增加97.7亿元和80.1亿元。

(二）抗击非典取得阶段性重大胜利。 面对突如其来的非典疫情，全市上下万众一心，众志成城，按照上级的决策部署，坚持依法防治、科学防治、群防群治，有效地切断了疫情的输入和传播渠道，确保了全市无疫情。坚持把防治非典与“双创”相结合，与开展爱国卫生运动、全民健身运动和科普教育活动相结合，与构建农民健康保障体系相结合，切实增强了疫病防治能力。同时，坚持一手抓防治非典，一手抓经济发展，及时采取应对措施，夺取了“双胜利”。

(三）改革开放不断深入。 招商引资扎实推进，全市到位外来固定资产投资65.3亿元。实际利用外资2.7亿美元，增长167.3%。成功举办了2003东营经贸洽谈会，取得了丰硕成果。东营经济开发区等各类园区呈现良好发展势头。对外经贸不断扩大，全市完成进出口总额8.4亿美元，增长61%，其中出口4.4亿美元，增长49.9%；对外经济技术合作完成营业额3.3亿美元，增长3.4倍。企业改革不断深化，全市98%的乡及乡以上国有、集体企业完成了产权制度改革，股权结构进一步优化。农村税费改革继续深化，停征了乡村公益事业金，取消了除海水养殖外的农业特产税。粮食、棉花流通体制改革有序推进。财税、价格、投融资等项改革不断深入。整顿和规范市场经济秩序工作取得新的成效。进一步整顿规范土地市场秩序，清理规范各类园区，严格执行土地“五统一”管理制度，对经营性用地全面实行了招标、拍卖和挂牌出让。事业单位改革稳步推进。

(四）城市和基础设施建设力度加大。 中心城专业规划、详细规划编制步伐加快，建成区控制性详规覆盖率达到100%；城市建设和改造进一步加强，城市功能不断完善；城市绿化、美化、亮化和环境综合整治取得较大成效，新增绿地面积465万平方米，城市面貌发生了新的变化；城市管理综合执法工作顺利实施，城市管理水平明显提高。县城和小城镇快速发展，全市城市化水平达到54%。村庄规划和改造也取得了一定成效。围绕完善立体交通、安全防护、引水供水体系和海洋渔业开发设施、“数字化东营”建设等方面，实施了一批重点基础设施项目，经济社会发展的基础条件进一步改善。

(五）区域经济社会一体化迈出新步伐。 油地军校团结发展的思想深入人心，相互支持、共同发展的氛围更加浓厚，凝聚起了推动区域经济社会发展的巨大合力。围绕共建美好家园，联合加大投入，加快了中心城的建设和改造。油地共同投资又兴建了一批重大基础设施，42公里的防潮大堤，顶住了50年一遇的特大风暴潮袭击，确保了中心城安全，谱写了又一曲油地结合的凯歌。油地共同兴建油田工业园初见成效。油田帮扶乡镇工作取得新的进展。制定完善政策措施，优化发展环境，深化安全文明油区创建活动，加大了对油军校改革和发展的支持力度。油军校的快速发展，又有力地带动和促进了地方的发展。

(六）社会全面进步。 思想道德建设不断深入，群众性精神文明创建活动广泛开展，“信用东营”建设取得较大成效，社会文明程度进一步提高。普法教育和依法治市扎实推进，依法行政水平有了新的提高。基层民主不断扩大，村民自治和社区建设得到加强。科教兴市战略深入实施，被命名为全国科技进步先进市和技术创新工程示范城市。基础教育、职业教育和成人教育全面发展，教育质量有了较大提高。加强公共卫生体系建设，提高了疾病预防控制和突发公共卫生事件的应急处理能力。文化、体育事业进一步繁荣，新闻出版、广播电视事业健康发展。双拥工作取得显著成效，被命名为全国双拥模范城。老龄、残疾人保障和妇女儿童工作受到全社会的重视。气象工作在防灾减灾中发挥了重要作用。外事侨务、民族宗教、人民防空、无线电管理、地名、史志、档案等各项事业都取得了新的成绩。加强污染综合防治，推进生态建设，环境质量明显改善。坚持开发与保护并重，国土资源管理工作得到加强。人口与计划生育工作水平不断提高，人口自然增长率3.8‰。认真做好群众来信来访工作，及时化解各类社会矛盾，加强社会和油区治安综合治理，深入开展“严打”整治斗争，毫不放松地抓好安全生产，有效地维护了社会稳定。

(七)“双十工程”建设取得显著成效。 在为民办10件实事方面，全面推行农民医疗保险制度，全市参保率达到98%，改造了33处乡镇卫生院，建设和改造了200处农村社区卫生服务站，强化农村卫生网络服务功能，实行药品集中招标采购和统一配送，初步构建起了农民健康保障体系；改造和扩建乡镇敬老院36处，“五保”老人集中供养率达到97%，市政府每年为每人发放200元生活补贴；建设“星光计划”示范点20处，实施了老年公寓和无障碍设施建设；建设学生食堂、宿舍9.3万平方米，解决了农村6万多名中小学生在校食宿问题；建设改造利民桥115座；改造中心城居住小区40个，建设改造中心城公厕75座，并对单位内部厕所进行了改造；改造城乡首集市场22处；黄河口旅游南线公路建设改造工程竣工通车，并对景点进行了规划设计；对3000多辆公交、出租、环卫车进行了压缩天然气改造；市直机关经济适用房建设一期工程完成楼房主体工程量的91%，二期工程已经铺开。在实施10大工程方面，顺利实现了“双创”目标，被命名为国家卫生城市和环境保护模范城市；建设改造城市主次干道39条、101.7公里；中心城水域综合治理任务圆满完成；超额完成了造纸示范林工程任务，造林面积13.8万亩；东营港扩建工程已经奠基；完成了渔港建设任务，增强了渔业生产服务和安全保障功能；实施了麻湾、宫家干渠改造和王庄二干延伸改造工程，在全省率先基本完成了大中型灌区节水改造任务，被授予全国水土保持生态环境建设示范城市；实施了黄河堤防道路硬化工程，改造建设了7条城乡骨干道路和896公里的农村公路；开通了“数字化东营”城域网；百姓平安工程也完成了年度建设任务。

(八）人民群众生活进一步改善。 城镇居民可支配收入达到10199元，增长11.5%；农民人均纯收入3510元，增长10.8%；城乡居民人均储蓄存款16260元，当年增加2568元。加强就业和再就业工作，新增就业3.4万人，其中再就业1.3万人；城镇登记失业率为2%。不断完善社会保障体系，“两个确保”保持100%，城乡“低保”做到了应保尽保。完善企业最低工资标准制度，积极解决拖欠劳动用工特别是农民工工资问题。加大了对弱势群体和困难群众的扶助力度。坚持统筹城乡发展，加大对“三农”的倾斜力度，大力改善农村生产生活条件。经过近年来的努力，在全省率先基本实现了农村“五通三保”，即村村通柏油路、通自来水、通客车、通有线电视、中小学微机联网校校通，农村最低生活保障、农民医疗保险和“五保”老人集中供养。

（九）政府自身建设得到加强。 进一步规范完善了市政府议事决策的程序和机制，对一些重大问题建立了公示和听证制度，提高了政府决策的科学化、民主化水平。转变政府职能，推进政府工作提速，提高了办事效率和服务质量。对市级行政审批事项进行了两次清理，取消46项、下放和分级管理57项、改变管理方式62项。同时进一步精简了审批环节，规范了审批行为，完善了一站式审批、一条龙服务和限时办结、服务承诺等制度。大力推行政务公开，对市政府文件和常务会议、市长办公会议研究事项通过政府公报和新闻媒体等向社会公开，提高了政府工作的透明度。进一步完善了行政效能监察机制。转变工作作风，完善督查考核办法，促进了各项工作落实。加强调查研究，制定了加快县域经济、服务业发展和支持油田存续企业改制等17项政策措施，推动了经济社会的发展。努力增强政府工作的预见性，在实践中探索创新，积累了新的经验。加强廉政建设，规范从政行为，纠正部门和行业不正之风及从源头上预防和治理腐败等项工作都取得了新的成效。坚持不懈地抓好软环境建设，进一步营造了亲商、富商、安商的氛围。

 各位代表!过去的一年，我们遇到的困难比预料的要大，取得的成绩比预料的要好。这是省委、省政府和市委正确领导的结果，是市人大及其常委会监督支持、人民政协和各民主党派参政议政的结果，是油地军校携手共建、全市人民团结奋斗的结果。在此，我代表市人民政府向全市人民和社会各界，向所有为东营的建设和发展做出贡献的同志们、朋友们表示衷心的感谢并致以崇高的敬意!

同时，我们也清醒地看到，经济社会发展中还存在不少困难和问题，政府工作中也存在缺点和不足。主要是，地方经济整体素质和竞争力还不强，发展的基础不够稳固；有些领域的改革措施不到位，经济发展中的体制性障碍仍然存在；县域经济总体发展水平还不高，有的县区和部分乡镇财政比较困难；城乡差距大，部分农村生产生活条件较差；高层次专业人才和适用型人才缺乏，劳动力整体素质有待提高；社会不安定因素还在不断出现，维护稳定的任务还很艰巨；干部队伍的思想解放程度、工作水平和驾驭能力与加快发展的形势还不相适应，政府工作效率和服务质量有待于进一步提高。对这些问题，我们一定高度重视，采取切实有效的措施，认真加以解决。

各位代表!2003年正值东营建市20周年。20年艰苦奋斗，20年沧桑巨变。东营已经实现了由荒凉落后到初步繁荣的历史性转变。前进的道路并不平坦，但任何艰难险阻都不能阻挡我们前进的步伐。我们务必更加振奋精神，更加扎实苦干，更加奋发有为，不断夺取新胜利，创造新辉煌!

二、今年政府工作的目标任务

今年是深入贯彻党的十六大和十六届三中全会精神，深化改革、扩大开放、加快发展的重要一年，也是实现“十五”计划的关键一年。国家宏观政策保持稳定，国际经济增长回升，经济社会发展的环境条件进一步改善。胜利油田正值“中兴”，进入了全面提升整体发展水平的新阶段。东营大地充满生机，全市经济社会进入了快速发展的新时期。总体上看，机遇大于困难，有利条件多于不利条件。只要我们抓住机遇，开拓进取，坚定信心，精心运筹，东营的大好形势就会不断巩固和发展。 今年政府工作的总体要求是：以邓小平理论和“三个代表”重要思想为指导，深入贯彻党的十六大和十六届三中全会精神，认真落实市委工作会议确定的工作思路，不断完善“五位一体”的发展路子，牢固树立全面、协调、可持续的科学发展观，以“五个统筹”统领经济社会发展全局，高度重视解决“三农”问题，加快推进新型工业化，大力发展现代服务业，努力扩大开放，不断深化改革，转变经济增长方式，优化发展环境，进一步激发各方面解放思想、干事创业、加快发展的积极性，凝聚合力，促进国民经济持续快速协调健康发展和社会全面进步，开创各项工作的新局面。

 今年政府工作的主要目标任务是：

----经济保持较快的增长速度，质量和效益有新的提高。全市国内生产总值增长13%，其中地方增长18%；全社会固定资产投资增长25%，其中地方增长40%；实际利用外资增长28%；外贸出口增长18%；社会消费品零售总额增长14%；地方财政收入增长13%。实际工作中要加倍努力，力争发展得更快更好。

——实施“五个统筹”取得新的成效，对外开放迈出更大步伐，县域经济实力明显增强，城市化进程进一步加快，油地军校结合更加深入，各项社会事业全面发展，人与自然更加和谐。

——各项改革扎实推进，经济体制和运行机制不断完善；科技进步明显加快，对经济增长的贡献率有较大提高，经济社会发展的活力和动力进一步增强。

——人民群众生活水平有新的提高，城镇居民可支配收入增长9%，农民人均纯收入增长6%，城镇登记失业率控制在2%以内。

三、集中精力抓经济促发展

（一）扩大招商引资和对外经贸。 坚定不移地抓好招商引资，坚持“三并举、三突出”的工作思路，不断提高招商水平。继续实行招商引资目标责任制，进一步调动全民招商的积极性。搞好专业招商队伍建设，提高人员素质，完善激励机制，加强专业招商。积极推动企业招商和油地校联合招商。紧紧抓住世界经济结构调整和产业转移的机遇，瞄准韩国、日本、台湾、香港等重点国家和地区，更加富有成效地开展招商活动，力求在境外招商上实现更大突破。加强与国外中介机构、我国驻外机构、外国驻华机构和华人华侨的联系，聘请一批招商代理，不断拓宽招商渠道。注重加强对外来投资企业的跟踪服务，使其依法获得较高回报，促进以商招商。举办2004东营经贸洽谈会，确保办出水平、办出成效。加强项目筛选论证，在坚持大中小项目一起引的同时，集中力量抓一批重大项目。切实抓好软环境建设，增强环境竞争力，努力使东营成为投资成本低、服务优、回报高，最适宜投资者创业发展的城市之一。大力发展对外经贸。适应出口退税机制改革的新形势，加快出口产品结构调整，大力发展加工贸易，扩大出口规模，提高出口效益和竞争力。深入实施市场多元化战略，加大国际市场开拓力度。认真落实鼓励扶持政策，壮大出口骨干企业，培育新的出口增长点。实施“走出去”战略，积极组织劳务输出，鼓励有条件的企业到境外承揽工程、投资办厂，跨国经营。

(二)高度重视并解决好“三农”问题。 把解决“三农”问题作为全局工作的重中之重，坚持统筹城乡经济社会发展，促进农业增效、农民增收、农村经济社会全面发展。第一，以资源开发带动农业结构调整。加大荒地、荒滩、荒水、浅海等资源开发力度，大力发展畜牧、水产、蔬菜、冬枣、速生林等优势产业。严格保护耕地，鼓励发展优质高效粮食生产，搞好粮食储备，确保粮食安全。同时大力发展农村二三产业，促进农民向非农产业和城镇有序转移，拓宽农民增收渠道。第二，推进农业产业化、标准化。继续加大扶持力度，培育一批规模大、带动力强的龙头企业。健全科技、信息、购销等农业社会化服务体系和农产品市场体系，积极发展农村专业合作经济组织，提高农民的组织化程度和开拓市场的能力。研究补贴办法，对购置和更新大型农机具给予一定补贴，提高农业机械化水平。进一步完善农产品检验检测体系，建设一批标准化生产基地，建成黄河三角洲无规定动物疫病区，提高农产品品质和市场竞争力。实施出口农产品“绿卡行动计划”，提高出口创汇能力。第三，加强农村基础设施建设。加快干渠、支渠、田间渠改造及骨干河道治理，进一步配套完善引、蓄、排、节水利体系。加强农村能源建设，发展农村沼气。实施农村绿化工程，进一步改善农村的生态环境。改造农村公路337公里，硬化村内主街道200公里。同时完善通讯、供电等基础设施。第四，认真落实党在农村的各项政策。稳定和完善土地承包关系，鼓励和规范土地承包经营权流转。完善土地征用补偿办法。深化农村税费改革，全部取消农业特产税，同时降低农业税税率2个百分点。放开粮食收购市场，把对粮食流通环节的间接补贴改为对农民的直接补贴。继续治理“三乱”，切实减轻农民负担。第五，加强农村劳动力培训。围绕发展农村优势产业和促进农村劳动力转移，搞好技能培训，增强农民致富本领。深入开展科技、文化、卫生“三下乡”活动，推动农村社会事业发展。

(三)加快加工制造业基地建设。 省政府确定在东营建设山东加工制造业基地，意义重大，影响深远。我们要进一步搞好规划，以园区为载体，以招商引资和现有企业膨胀为途径，以科技进步为动力，打造“东营制造”品牌，努力加快加工制造业基地建设步伐。要进一步搞好东营经济开发区等园区规划建设，加大基础设施配套力度，大力提高承载能力。要建立多元投入机制，保持强劲投入力度，加快化工、造纸、纺织、橡胶等主导产业的发展。今年全市地方工业固定资产投入力争达到100亿元以上。要大力推进技术创新，瞄准世界先进水平，加快运用高新技术和信息化手段改造传统产业，积极发展电子信息、新材料、精细化工、生物技术与制药等高新技术产业，提升制造业水平。要加强区域经济和骨干企业发展战略研究，引导企业在明确发展方向的基础上，不断加大投入，改善管理，提高核心竞争力和市场占有率，快速做大做强。要关心企业家的成长，使其不断开阔视野，增强创业意识，提高经营管理能力，努力造就一支高素质的企业家队伍。

(四)大力发展服务业。 发展服务业有利于增加就业岗位，有利于增加财政收入，有利于改善投资环境。要把发展服务业摆上战略地位，放宽市场准入，积极给予扶持。要搞好规划，加大投入，改造提高商贸流通等传统服务业，大力发展信息、现代物流、社区服务、中介服务、旅游等新兴服务业。今年重点规划建设“四城”、“两市”、“两街”、“两中心”等一批大型经营设施。要整合物流资源，规划建设物流园区，加快现代物流业发展。要加大旅游资源开发力度，搞好景区景点建设，从今年起，重点实施黄河口生态旅游、滨海旅游、油城旅游、文化旅游“四大工程”，努力打造以“神奇黄河口、梦幻石油城、生态大观园”为特色的旅游品牌。

(五)加强城市和基础设施建设。 深化城市发展战略研究，进一步明确我市在山东半岛城市群中的发展定位。坚持规划先行，进一步搞好中心城详细规划和专业规划编制，提高规划编制水平和覆盖率。推进西城改造，搞好东城和东西城对接区建设。年内建设改造城市主次干道27条，基本完成主次干道建设改造任务，同时配套完善供水、污水处理、城市生活垃圾无害化处理等市政公用设施。提高设计水平，建设精品工程，丰富城市文化内涵，提高城市品位。巩固“双创”成果，搞好城市绿化、亮化、美化，启动森林公园建设，进一步展现大空间、大绿地、大水面的独特城市风貌，努力把东营建设成为最适宜人类居住的城市之一。建立长效机制，严格依法管理，促进城市管理再上新水平。完善投融资机制，为城市建设筹集更多资金。大力推进县城和小城镇规划建设，同时加强村庄规划建设和环境整治。继续加大基础设施建设力度。加快东营港扩建和广利商港建设；配套完善东营机场，争取增加航线和飞行班次；推进环渤海高速公路建设，争取实施黄东大铁路建设，进一步构建海陆空立体交通体系。搞好以防潮堤为重点的黄河三角洲安全防护体系建设。做好大型火力发电厂的前期工作。建设公用热电联产项目，推进中心城和县城集中供热。进一步加强通讯、“数字化东营”等设施建设。

(六)切实做好财税金融工作。 大力培植财源，加强税收征管，完善社会综合治税机制，做到应收尽收。按照“五个统筹”的要求调整优化支出结构，大力压减一般性支出，集中财力办大事。在保工资和法定支出的基础上，继续加大对“三农”、社会事业、社会保障、弱势群体和困难群众的倾斜力度，逐步建立起公共财政框架。今年预算内外资金计划用于“三农”的达41亿元，用于社会事业的达4亿元，用于社会保障和对弱势群体、困难群众扶助的9191万元，均比去年有较大幅度增长。加快预算编制改革，全面推行部门预算。强化预算约束，加强审计监督，提高财政资金使用效益。完善财政集中支付和政府采购制度。严格执行“收支两条线”管理规定，把预算外资金全部纳入统一管理。支持金融机构加快改革与发展，不断完善信用担保体系，增强融资能力。搞好农村信用社改革，将其真正办成由农民、农村工商户和各类经济组织入股，为“三农”服务的社区性地方金融机构。

 (七)深化经济体制改革。 进一步推进企业产权制度改革，年内全部完成改制任务。引导企业调整股权结构，完善法人治理结构，健全激励约束机制。积极做好企业上市工作。大力支持油田存续企业改制，增强发展活力。深化国有资产管理体制改革，完善国有资产经营管理机构，建立健全国有资产监督管理体系。依法加强集体资产的经营管理，积极探索建立农村集体经济的规范化、公司化管理体制。进一步完善市场体系，培育提升商品市场，规范发展土地、技术、劳动力等要素市场。实行行政执法、行业自律、舆论监督、群众参与相结合，进一步整顿和规范市场经济秩序。继续搞好计划、价格、投融资等改革。积极稳妥地推进事业单位分类管理和改革。搞好供气、供热、绿化、环卫等公用事业改革。改革通讯费、取暖费等管理办法。

(八)积极实施科技强市和人才兴市战略。科技是第一生产力。要加快科技推广体系建设，努力推动高新技术和适用技术在经济社会各个领域的广泛应用。强化企业技术创新主体地位，抓好企业研发中心建设，深化产学研结合，加大政府科技投入，加快建立区域技术创新体系。办好高新技术产业开发区，搞好高新技术企业孵化器建设，实施好高技术产业化示范项目，大力引进、开发和转化高新技术成果。鼓励发展民营科技企业和民营科研机构。

国以才立，政以才治，业以才兴。经济的竞争归根到底是人才的竞争。要牢固树立人才资源是第一资源的观念，紧紧抓住培养、引进、用好三个环节，着力建设行政人才、企业经营管理人才和专业技术人才三支队伍。多层次、多渠道、大规模开展人才培训，造就一批高层次、高技能人才。充分发挥东营职业学院、高级技工学校、胜利职业学院等院校的作用，培养更多的专业技术人才。创新人才工作机制，大力引进经济社会发展需要的人才。努力为各类人才施展才华创造条件，形成优秀人才脱颖而出、人尽其才、才尽其用的生动局面。

(九)推进区域经济社会一体化和县域经济发展。 深化油地军校结合，进一步凝聚发展合力。在思想上融合，高举团结发展的旗帜，真心实意地支持胜利油田实现“油气当量重上三千万、存续经济总量翻一番、职工生活水平要有新提高”三大战略目标，真心实意地支持石油大学加快发展，真心实意地支持济军生产基地调整改革。在资源上整合，挖掘区域科技、人才等资源潜力，使各类资源得到有效充分利用。在发展上统筹，推进油田工业园建设，统筹发展接续替代产业；统筹城市建设，共同加大投入，共建美好家园；统筹规划建设重大基础设施，加快区域经济一体化、城市规划建设管理一体化、基础设施建设一体化步伐。进一步做好油田帮扶乡镇工作。油地军校结合蕴藏着巨大的潜力，结合出生产力、出竞争力。油地军校结合的不断深入，必将极大地促进我市的繁荣发展，必将创造出惠及全市人民的幸福美好生活!

县域经济是统筹城乡发展的结合点，在地方经济中具有举足轻重的地位。要立足实际，理清思路，发挥优势，大力发展特色经济，不断壮大县域经济综合实力。创造更好条件和更优环境，加快民营经济发展，使其成为县域经济的主体、县乡财政的支柱。深化县域综合改革，增强发展活力。落实促强扶弱政策，促进经济强镇率先发展，实现强者更强，促进经济欠发达乡镇跨越发展，逐步缩小差距。

(十)不断改善人民群众生活。 全面增加城乡居民收入，让人民群众共享经济发展成果。就业是民生之本。要认真落实扶持就业和再就业的各项优惠政策，坚持劳动者自主择业、市场调节就业和政府促进就业的方针，积极开发就业岗位，拓宽就业渠道。加大再就业投入，扩大培训范围，完善公益性岗位储备制度，重视解决“4050”人员的再就业问题。加强劳动力市场信息网络建设，进一步完善就业服务体系。搞好大中专毕业生就业指导。加强对企业用工行为的规范和监督，切实解决拖欠劳动用工特别是农民工工资问题，保护劳动者的合法权益。健全社会保障体系，继续巩固“两个确保”，依法加强社保基金征缴，扩大基本养老保险、失业保险和基本医疗保险覆盖面，加快推进企业退休人员社会化管理服务。切实做好城乡低保工作。健全农民医疗保险和大病救助制度，建立失地农民社会保障制度和农村养老保险制度。完善农村“五保户”集中供养机制，市政府对“五保”老人的生活补贴由每人每年200元提高到400元。完善社会救助制度，加大对弱势群体和贫困人口的扶助力度。加快经济适用房建设，搞好小康示范村建设试点，改善城乡居住条件。建立完善城市社区管理和服务体系，搞好服务设施建设，积极开展面向社区居民的家政、养老、托幼、帮困、助残等项服务。巩固农村“五通”成果，加强维护管理，搞好配套完善.

四、推动社会全面进步

加强精神文明建设。深入开展“三个代表”重要思想宣传教育活动，进一步加强和改进思想政治工作。认真贯彻公民道德建设实施纲要，加强社会公德、职业道德和家庭美德教育。广泛开展创建文明城市、创建小康文明村活动，提高市民的现代文明素质。扎实推进“信用东营”建设，加强诚实守信教育，完善社会信用体系，建立健全信用评估、信息披露、监督和失信惩戒等机制，进一步塑造东营“文明立市、诚信兴业”的良好形象。

繁荣发展各项社会事业。 进一步提高义务教育水平，积极推进学前教育和高中教育的普及，大力发展职业教育、成人教育。全面推进素质教育，增强受教育者的就业能力、创新能力和创业能力。积极扶持农村教育发展。深化卫生管理体制改革,加强公共卫生设施建设，完善传染病预防与救治网络，增强突发性公共卫生事件应急处理能力。加强农民健康保障体系建设，进一步增强农村卫生服务功能。深化文化体制改革，规范文化市场秩序，加强文化设施建设，鼓励发展文化产业，丰富城乡文化生活。搞好全市广播电视网络的整合开发。开展全民健身运动，提高全民健康水平和竞技体育水平。办好第七届市运会。坚持不懈地做好人口和计划生育工作，积极推进计划生育综合改革和优质服务，全市人口自然增长率控制在9‰以内。依法加强土地管理，合理开发利用各种资源。积极开发地热资源，发展城市供热等项目。制定实施生态市建设规划，加强生态建设与保护，严把建设项目环境管理关，加大污染源治理力度，搞好黄河三角洲自然保护区和地质公园建设，大力发展高效生态经济和循环经济，增强可持续发展能力。关心支持妇女儿童、残疾人事业，做好老龄工作。加强全民国防教育，抓好民兵预备役建设，巩固扩大双拥共建成果。进一步做好档案、气象、民族宗教、对台、外事侨务、地名、史志、无线电管理、人防、防震和减灾救灾等工作。

加强民主与法制建设。 各级政府要自觉接受人大及其常委会的监督，支持人民政协履行职责，加强与各民主党派、工商联、无党派人士的联系，充分发挥工会、共青团、妇联等人民团体的桥梁纽带作用。完善基层群众自治性组织，保证人民群众依法行使民主权利。进一步完善重大问题的议事规则和决策程序，建立健全人民建议征集、重大事项社会公示、社会听证和专家咨询等制度。深入开展普法教育，不断推进依法治市。认真贯彻实施《行政许可法》，提高依法行政水平。加强执法监督，强化执法责任制和执法过错追究制，做到严格执法、公正执法、文明执法。健全完善法律援助体系，依法保障弱势群体的合法权益。

大力推进“平安东营”建设。 坚持工作重心下移，加强基层基础工作，及时排查、调处社会矛盾纠纷，切实做好信访工作，努力预防、化解群体性、突发性事件。深入开展严打整治斗争，全面落实社会治安和油区治安综合治理各项措施。坚持不懈地抓好安全生产工作，认真落实安全生产责任制和责任追究制，加大投入，强化监督检查和事故隐患整改，完善事故预警和救援机制，确保全市安全生产形势持续稳定。

各位代表!在全面推进各项工作的同时，我们将围绕人民群众生产生活的需要，围绕建设山东加工制造业基地的需要，围绕创建文明城市和建设生态市的需要，着力为民办好农村沼气建设、农民培训和“科技下乡”服务体系建设、“五保”老人集中供养长效机制建设、粮食储备体系建、创建山东省公共卫生安全示范区、实施援助农村教育计划等十件实事，实施好东营港扩建工程、水利“三百利民”工程、农村绿化工程、城乡基础设施建设改造工程、农村有线电视普及和数字化工程等十大工程。对这十件实事和十大工程，我们要实行严格的责任制，强化督查调度，确保说到做到，按期完成，取信于民!

 五、切实加强政府自身建设

新的形势和任务对政府工作提出了新的更高的要求。各级政府必须坚持为民、务实、清廉，不断加强自身建设，更好地担当起历史和人民赋予的重任。

要大力提高政府工作效率。 推进政府工作提速，提高工作效率，是改进政府工作的重中之重，也是提升环境竞争力的关键环节。为此，市政府把今年确定为政府效率年，全面提高管理效率、办事效率和抓落实的效率。深化行政管理体制改革，转变政府职能和管理方式，逐步把政府职能转到经济调节、市场监管、社会管理和公共服务上来，努力建立起规范高效的新型行政管理运行机制。结合贯彻实施《行政许可法》，进一步深化行政审批制度改革，精简审批事项，减少审批环节，规范审批行为，提高审批效率。认真落实政务公开、服务承诺、限时办结等制度，完善一站式审批、一条龙服务机制，努力为市民和外来投资者提供优质快捷的服务。进一步精简会议、文件，减少不必要的应酬活动，集中精力谋全局、抓大事。进一步完善决策目标、执行责任、考核监督“三个体系”，加强政务督查考核，抓好各项工作的落实。加大行政效能监察力度，促进行政效能和服务质量不断提高。

 要切实保护好、引导好和发挥好各方面解放思想、干事创业、加快发展的积极性。 要鼓励支持创新，以创新观念为先导，创新思路、创新体制、创新机制，使政府的各项工作更好地体现时代性、把握规律性、富有创造性。要倡导调查研究，研究新情况，推出新举措，解决新问题。干事创业是一种境界、一种精神，也是一种能力。全体政府工作人员特别是领导干部要重视学习，勤于学习，善于学习，把理论学习与实践探索结合起来，不断提高科学判断形势的能力、驾驭市场经济的能力、应对复杂局势的能力、总揽全局的能力和解决实际问题的能力。要鼓励创新者、支持干事者、鞭策落后者，使我们的干部队伍更加朝气蓬勃，使我们的事业充满生机和活力。

要坚持不懈地加强廉政建设。 坚持从严治政，认真落实党风廉政建设责任制，进一步抓好领导干部廉洁自律，严肃查处违纪违法案件，坚决纠正部门和行业不正之风。建立健全教育、制度、监督并重的惩治和预防腐败体系，加大从源头上防治腐败的力度。加强思想道德教育和纪律教育，筑牢拒腐防变的思想防线；加强廉政制度建设，建立反腐倡廉的制度体系；加强监督制约，形成权力正确行使的有效机制。全体政府工作人员特别是领导干部都要自觉加强世界观改造，树立正确的权力观、地位观、利益观，始终保持谦虚谨慎、不骄不躁的作风，始终保持艰苦奋斗的作风。

 要牢固树立正确的政绩观。 树立正确的政绩观，最根本的是牢记全心全意为人民服务的宗旨，把维护好、实现好、发展好最广大人民群众的利益作为最大追求。全体政府工作人员要始终不渝地坚持权为民所用，堂堂正正做人，清清白白为官，扎扎实实办事，用人民赋予的权力服务于人民，造福于人民；始终不渝地坚持情为民所系，倾听群众呼声，关心群众疾苦，时刻把人民群众的安危冷暖挂在心上；始终不渝地坚持利为民所谋，时刻把人民利益放在首位，从群众最关心、最迫切要求解决的实际问题入手，诚心诚意办实事，尽心竭力解难事，坚持不懈做好事。要求真务实，力戒形式主义、官僚主义，使经济得到实实在在的发展，让群众得到实实在在的利益。

 各位代表!人民群众是我们的力量源泉。只要我们一切为了群众，一切依靠群众，与群众心连心、同呼吸、共命运，脚踏实地工作，俯首为民办事，就会赢得广大人民群众的信赖和支持，就会无坚不摧、无往而不胜!

各位代表!我们在全面建设小康社会的伟大征程上已经迈出了坚实的一步。回顾过去，豪情满怀；展望未来，信心倍增。让我们紧密团结在以胡锦涛同志为总书记的党中央周围，高举邓小平理论和“三个代表”重要思想伟大旗帜，在市委的坚强领导下，解放思想，抢抓机遇，同心同德，干事创业，为实现全面建设小康社会的宏伟目标而努力奋斗!
